# A Cross-Sectional Study of Risk Factors for Irritable Bowel Syndrome in Children 8–13 Years of Age in Suzhou, China

**DOI:** 10.1155/2014/198461

**Published:** 2014-05-11

**Authors:** Xueping Zhu, Weichang Chen, Xiaoli Zhu, Yueping Shen

**Affiliations:** ^1^Department of Neonatology, Soochow University Affiliated Children's Hospital, Suzhou 215003, China; ^2^Department of Gastroenterology, The First Affiliated Hospital of Soochow University, Suzhou 215006, China; ^3^Department of Intervention, The First Affiliated Hospital of Soochow University, Suzhou 215006, China; ^4^Statistics Office, Soochow University, Suzhou 215000, China

## Abstract

To determine the prevalence and risk factors of IBS in children 8–13 years of age in Suzhou city, a cross-sectional study was conducted on children in grades 1 through 6 in public elementary schools in three districts of Suzhou. A multistage stratified random-sampling survey was conducted in a primary investigation using standardized questionnaires. Rome II criteria were used to confirm IBS and their risk factors were analyzed. Of 8,000 questionnaires 7,472 responded satisfactorily for a response rate of 93.4%. IBS was diagnosed in 10.81%. A decrease in the prevalence of IBS was significantly associated with advancing age and grade in school (trend test, *P* < 0.05). The prevalence of IBS in females was higher but not significantly different than males. The significant risk factors for IBS included young age (OR = 0.94), food allergy (OR = 1.53), gastroenteritis during childhood (OR = 1.29), eating fried food (OR = 1.62), anxiety (OR = 1.49), psychological insults in early childhood (OR = 1.47), and parental history of constipation (OR = 1.81; all *P* < 0.05). IBS prevalence of 10.81% in study population warrants preventive measures such as encouraging dietary changes, preventing gastroenteritis and childhood psychological insults.

## 1. Introduction

Irritable bowel syndrome (IBS) is a functional disease of the intestine, characterized by abdominal discomfort or abdominal pain with or without changes in bowel habits. The prevalence of IBS in adults varies across the globe: −10% to 15% in Europe and North America (by Rome criteria II), 11% to 17% in Oceania (by Manning and Rome criteria II), about 10% in Africa (by modified Manning criteria), and about 5% in Asia (by Manning and Rome criteria II) [1–3]. Recently, an increasing prevalence of IBS has been reported in Asia: −8.6% and 9.8% in Singapore and Tokyo, respectively [4]. In China, there has been a substantial volume of research on IBS in adults. Studies in Beijing and Guangdong reported different prevalence rates [5, 6], indicating variations among geographical locations. The prevalence of IBS in cities in China is about 10.5%, second only to prevalence of the common cold. In gastroenterological clinics, the IBS accounts for 20–50% of all visits [7]. Although not life-threatening, IBS is a disease causing heavy economic burden due to increased work or school absenteeism and impaired quality of life in affected people as well as increased use of health care services [8–10]. IBS places huge financial burden on patients and their families; the total cost of treatment has been estimated as high as $3 billion in the United States [11].

There are a few epidemiologic studies on IBS in children, but few are from China. Epidemiologic studies in populations less than 18 years of age suggest higher prevalence among teenagers and children compared to adult population [12–16]. Miele et al. [17] reported that, in a study of 9,660 children less than 12 years of age who were observed for three months, 194 met the criteria for IBS based on Rome II criteria and 27 (13.9%) of those meeting criteria for IBS were eventually diagnosed with IBS. Walker et al. [18] reported recurrent abdominal pain as the common symptom in 44.9% of children with IBS. The prevalence of IBS in teenagers and children varied between Shanghai and Heilongjiang, 11.72% in Shanghai and 14.02% in Heilongjiang [19–21]. A large-scale epidemiologic survey of IBS in children either abroad or from China is lacking. Therefore, we conducted an epidemiologic survey of children in grades 1 through 6 in elementary schools in Suzhou between November 2006 and March 2007. Our goal was to investigate the prevalence of IBS and identify risk factors for future diagnosis and prevention.

## 2. Materials and Methods

### 2.1. Methods

An epidemiological cross-sectional study is conducted.

### 2.2. Participants and Sample Size

This study used a stratified random-sampling survey with multistage clusters of children in grades 1 through 6 in the public elementary schools of Canglang, Pingjiang, and Jinchang urban districts in Suzhou from November 2006 to March 2007. Using the general formula *n* = *t* ^2^ *pq*/*d* ^2^ and data from a preliminary investigation [22] where *t* = 1.96 (error of the first kind), *p* = 13.25% (estimated prevalence), *q* = 1 − *p*, and *d* = 15% × *p* (permissive error), eight schools in Canglang district (four classes in each of the grades 1 through 6), five schools in the Jinchang district, and 14 schools in the Pingjiang district were surveyed. The study was approved by the Medical Ethics Committee of Children's Hospital Affiliated Soochow University and Suzhou Health and Education Bureau.

Before the study, we explained the purpose of the research project to the principals and teachers from each of the target schools, and the participants' parents were also informed through a letter. Members of the Suzhou Education Bureau joined specially trained doctors and graduates of the gastroenterology and the pediatric departments to train teachers in charge of the selected classes to administer the questionnaire. Students of grades 4–6 completed the self-reported questionnaires under the guidance of trained teachers, and the other students' (grades 1–3) questionnaires were filled by the trained teachers through phone-call to the participants' parents. Data in all returned questionnaires were carefully checked and the reliability was established as 97.2%.

The self-administered, standardized validated questionnaire for IBS in children was based on previous studies [19], psychological assessment in patients with IBS in Chinese version [23], and Rome II criteria [24]. It surveyed personal sociodemographic data, birth history, feeding methods during infancy, gastrointestinal symptoms during the neonatal period (including spitting, hiccups, difficulty defecating, dyspepsia, and intestinal infection), food habits (including cellulose, protein, fat, spicy, and fried foods), diagnostic criteria (Rome II criteria), evoking agents (including gastrointestinal infection, abuse of antibiotics, and analgesics) in infants and children, psychosomatic symptoms (including insomnia, fatigue, emotions, and back pain), family history (e.g., parental IBS, bowel problems, and depression), psychological history (anxiety, depression, and psychological insults), and surgical procedures. We used the Chinese version of SCARED to assess child anxiety-related emotional disorders [25] and the psychological insults included physical abuse, neglect, single family, and sexual abuse. Other information collected included name, age, body weight, parental income, gestational age at birth, birth weight, history of resuscitation at birth, breast feeding, food allergies (e.g., milk products, egg, seafood, peanuts, etc.), intestinal infections, gestational conditions of their mothers, and extra-abdominal symptoms.

### 2.3. Definition of Irritable Bowel Syndrome (IBS)

IBS includes intermittent or continuous symptoms, such as abdominal pain, abdominal distension, changes in bowel habits (diarrhea or constipation), and changes in stool texture (liquid stool, stool with mucus, and hard stool). For a diagnosis of IBS to be made, the subject needed to have at least two of the following three symptoms: (1) symptom relief with defecation and/or (2) onset associated with a change in frequency of stool and/or (3) onset associated with a change in appearance of stool [26]. Based on the Rome II criteria, the abdominal discomfort or pain should have been there for at least 12 weeks, not necessarily consecutive, in the preceding 12 months [27–30]. A total of 8,000 questionnaires were administered and 7,472 satisfactory responses were collected for a response rate of 93.4%. In the preliminary survey, IBS was suspected in 912 students and the suspected students were suggested to visit a physician to exclude organic disease. Those suspected of having IBS were subjected to a secondary interview and consultation that were based on Rome II criteria. The diagnosis of IBS was confirmed in 808 students (10.81%) after excluding organic diseases such as major abdominal surgery, peptic ulcer, ulcerative colitis, diabetes mellitus, hyperthyroidism, or mesenteric lymphadenitis.

### 2.4. Statistical Analysis

Questionnaires were coded twice and a database was established with Epidata 3.0 (Chinese Version); data entry was twice verified. The processed database was transformed into SAS datasets and analyzed further using SAS 8.0 statistical package (SAS Institute, Inc., Cary, NC). Chi-square test and stepwise multivariate logistic regression were used for analyses (entry and exclusion criteria: *α* = 0.05). Associations between risk factors and IBS were expressed as odds ratio (OR) with 95% confidence intervals (CI). All tests were double-sided, and the level of significance was set at *P* < 0.05.

## 3. Results

### 3.1. Prevalence of IBS

The ages of 7,472 students in the study ranged from 8 to 13 years, with the mean age of 1.90 ± 1.86 years; the ratio of male to female was 1.10 : 1. The prevalence of IBS in elementary school students in grades 1–6 in Suzhou was 10.81% and the prevalence of IBS in relation to age, gender, family income, gestational age, birth weight, and current weight is shown in Table 1. The prevalence of IBS decreased significantly with increasing age (Cochran-Armitage trend test: *P* < 0.05). The highest prevalence (13.40%) was observed in children 8 years of age and the lowest prevalence (9.40%) was in children 13 years of age. IBS was more prevalent in girls (11.30%) than in boys (10.30%), although the difference was not statistically significant (*P* > 0.05). There was no significant association between the prevalence of IBS and family income, gestational age, or birth weight. Children with birth weight larger than 4 kg had a marginal lower prevalence of IBS than those with normal birth weight and low birth weight (7.70% versus 11.20% and 11.50%, *P* = 0.09).

### 3.2. Multivariate Logistic Regression Analysis of IBS Risk Factors

The prevalence of IBS was significantly associated with food allergy, intestinal infection in the newborn period, gastroenteritis in childhood, fried food intake, anxiety, parental constipation, and psychological insults in childhood after using univariate logistic model.

Males and females had similar risk factors for IBS. Therefore, we used stepwise multivariate logistic regression with the entry and exclusion criteria (*α* = 0.05) to identify risk factors in pooled data from males and females. The possible risk factors for IBS among elementary school children in Suzhou included young age, food allergies, gastroenteritis during childhood, preference for fried foods, anxiety, psychological insults in childhood, and parental constipation (Table 2). The odds ratio of IBS for children with parental history of constipation was 1.81 (95% CI: 1.46–2.24) when compared with the control group. Children with food allergies were more likely affected with IBS than those without such allergies (OR = 1.53; 95% CI: 1.13–2.07). Anxiety and a history of psychological insults also significantly increased the likelihood of IBS (OR = 1.49; 95% CI: 1.16–1.93; OR = 1.47; 95% CI: 1.02–2.20, resp.).

## 4. Discussion

The present study, in a large sample size, has identified the prevalence of IBS in elementary school students in grades 1–6 in Suzhou as 10.81%. The prevalence was higher in females than in males and decreased significantly with increasing age and grades in school.

IBS is a disease of global significance. The prevalence of IBS in western countries is higher than in Asia. Studies on IBS in adults outside China have addressed causes, mechanisms, and risk factors; studies of the pediatric population suggest relatively high incidence. An epidemiologic survey by Harvard Medical School in the USA involving 507 school students reported IBS symptoms in 14% of high school students and 6% of junior high school students based on Rome II criteria [12]. The prevalence was higher in females than in males and higher in whites than in blacks. Together, these observations suggest that IBS is more common in older children and varies according to race and sex.

Rasquin et al. [31] used the Rome II criteria to diagnose IBS in 0.2% of children (mean age 52 months) seen by primary care pediatricians and in 22–45% of children 4–8 years of age presenting to tertiary care clinics. The main difference between the pediatric Rome III and II criteria was that the duration of abdominal pain was reduced from 3 months to 2 months [16]. In Istanbul, the prevalence of IBS in children 4 to 18 years of age using Rome III was 22.6%, which was higher than that detected using the Rome I and II criteria [16], similar to another study in Sri Lanka [15]. A simple stratified survey in Shanghai and Heilongjiang revealed higher IBS prevalence according to the Rome III criteria in high school students (17.80%) than in students of lower grades (11.45% for junior students, 13.53% and 12.37% for primary students) and significant differences in prevalence between those regions [32]. Shen et al. [33] found that the prevalence of IBS in first-year college students was 15.7% (14.5% and 16.8% for males and females, resp.). Zhou et al. [21] found incidences of IBS, functional constipation, and diarrhea of 19.89%, 24.93%, and 5.42%, respectively, in teenagers from Shanghai, Putong, and Jiading districts [20]. Gastrointestinal tract infections, abuse of pain-killers, and depression contributed significantly to IBS (*P* < 0.05), while fried food, air swallowing termination of hiccups, anxiety, and depression mainly contributed to functional constipation. IBS among 2,013 students aged 10–18 in Jiading district revealed an incidence of IBS of 20.72%, which increased with age. More girls than boys, especially in the older age group, are likely to be depressed and have IBS, indicating a link between depression and IBS and emphasizing the importance of having a healthy environment in both family and school [21].

The prevalence of IBS in elementary school students in grades 1–6 in Suzhou was 10.81%. Females had a higher prevalence than males, although the difference was not statistically significant (*P* > 0.05), similar to the study by Reshetnikov et al. [13]; however, Drossman [34] reported that females are more likely than males to be diagnosed with IBS significantly, inconsistent with our study, possibly due to the levels of sex hormones.

Studies in adults have suggested different reasons for varying prevalence of IBS between males and females. The inhibitory effect of the hypothalamus-pituitary reflex on ovarian hormones could improve the symptoms of IBS. Substituting leuprolide acetate for gonadotropin-releasing hormone (GRH) is useful for the treatment of IBS symptoms [35]. IBS symptoms are exacerbated during menstrual cycles in females [36]. Students of young age and lower grades (grades 1–6 in the elementary schools) had not reached sexual maturity and, consequently, the levels of sex hormones are not the same as in adults. Although some of the students in higher grades in the elementary school were already approaching puberty, their hormonal levels were not stable. This might explain our observation that although females had a higher prevalence of IBS than males, it was not statistically significant.

This study has demonstrated that prevalence of IBS decreased with increasing age and grades in school (*P* < 0.05); these results differ from those of Li et al. [32]. This discord might be the result of the large age range in their study population and the inclusion of teenagers, as well as larger age intervals for their samples. We found that the prevalence of IBS was not significantly associated with gestational conditions, birth history, birth weight, or family income, but children with birth weight >4 kg had a lower risk of being affected with IBS. The precise reason for this observation was not clear, but this observation was suggestive of a possible association between in utero development and IBS. Bengtson et al. [37] reported a possible link between lower birth weight (<1500 g) and developing IBS in the future from a twin study. The reason for such an association was fetal malnutrition. To some extent, our results agree with this observation.

Bytzer et al. [38] found relationships between low socioeconomic status and poor hygiene with IBS; however, we did not find a significant association between economic status and the prevalence of IBS. This might be because all children were from Suzhou and most of the subjects had no siblings. Although family income was not uniform across our study group, we believed that the disparity was not great enough to exert a significant influence on the quality of life of the children. IBS was associated with a history of food allergies, history of intestinal infection in the newborn period, history of gastroenteritis during childhood, preference for fried food, anxiety, parental history of bowel problem, and history of psychological insults during childhood in both sexes. Other authors outside of China have reported similar findings [32, 39, 40]. IBS was not associated with the history of antibiotic abuse or frostbite. Separate analyses for males and females found similar profiles of risk factors. The results of univariate logistic regression in males demonstrated that the history of sleep disturbance and back pain was associated with IBS in males but not in females. Based on data pooled from males and females, the risk factors for IBS among elementary school students in Suzhou included young age, history of food allergy, history of gastroenteritis during childhood, preference for fried food, anxiety, history of psychological insults during childhood, and parental history of bowel problem. Children with parental history of constipation were 1.81 times more likely to be diagnosed with IBS than control group children (95% CI: 1.46–2.24). Children with history of food allergies were more likely to be diagnosed with IBS than those without (OR = 1.53; 95% CI: 1.13–2.07). Anxiety and history of psychological insults during childhood also increased the probability of being affected by IBS (OR = 1.5, *P* < 0.05), similar to results observed in college students [33]. The pathogenesis of IBS appears to be multifactorial and interaction of the following factors likely plays a central role in the pathogenesis: heritability and genetics, dietary factors and intestinal microbiota, low-grade inflammation, and disturbances in the enteric nervous system (NES) of the gut [41]. Our study findings support this view.

There were certain limitations in our study. We did not analyze the subtypes of IBS in detail. The possible association between IBS and the degree of anxiety and subtypes of IBS and parents' definite bowel problem could not be derived.

In conclusion, this study demonstrated that IBS is a common disease in elementary school children in Suzhou. Possible risk factors of IBS included young age, food allergies, gastroenteritis during childhood, preference for fried food, anxiety, psychological insults during childhood, and parental constipation. To decrease the prevalence of IBS in children of this age group, more attention should be given to preventing gastroenteritis, encouraging dietary changes, and preventing childhood psychological insults.

## Figures and Tables

**Table 1:** Demographic characteristics-specific prevalence of IBS in children.

	Number of cases	(%)	Total surveyed *n* ^†^	*z*/*χ* ^2^-value	*P* value
Age (years)					
8	50	13.40	374	2.796*	0.0026
9	106	12.70	832		
10	171	11.20	1533		
11	191	10.17	1883		
12	207	10.20	2025		
13	74	9.40	784		
Sex					
Male	403	10.30	3917	2.015	0.15
Female	402	11.30	3555		
Annual family income (RMB)					
≤12,000	117	12.00	979	3.84	0.27
12,001–20,000	75	9.90	758		
20,001–36,000	108	9.60	1129		
>36,000	64	11.30	567		
Gestational age (weeks)					
≤35	25	10.30	244	0.712	0.87
35^+1^–37	68	10.50	651		
37^+1^–40	214	11.20	1904		
>40	48	10.20	471		
Birth weight (kg)					
<2.5	86	11.20	765	4.77	0.09
2.5–4.0	537	11.50	4671		
>4.0	28	7.70	362		
Weight (kg)					
≤30	250	11.59	2157	6.55	0.25
30.1–35	164	11.29	1453		
35.1–40	121	10.34	1170		
>40	161	10.23	1574		

*Cochran-Armitage trend test.

^†^Due to missing cases, the total number is less than 7472.

**Table 2:** Multivariate logistic regression analysis of risk factors of IBS in children.

Variables	Regression coefficients (*β*)	Standard error (*s*_*β*_)	Wald chi-square	*P* value	AOR (95% CI)
Intercept	−1.5013	0.3301	20.6802	<0.0001	—
Age (continuous variable)	−0.0596	0.0301	3.9178	0.047	0.94 (0.89–0.99)
History of food allergy	0.42	0.16	4.47	0.03	1.53 (1.13–2.07)
Childhood					
History of gastroenteritis	0.26	0.13	3.92	0.04	1.29 (1.00–1.63)
Preference for fried food	0.48	0.09	5.03	0.02	1.62 (1.34–1.96)
Anxiety	0.40	0.13	4.11	0.04	1.49 (1.16–1.93)
Parental history of constipation	0.59	0.11	5.83	0.01	1.81 (1.46–2.24)
History of psychological insults in childhood	0.40	0.19	4.21	0.04	1.47 (1.02–2.20)

## References

[1] Lule GN, Amayo EO (2002). Irritable bowel syndrome in Kenyans. *East African Medical Journal*.

[2] Saito YA, Schoenfeld P, Locke GR (2002). The epidemiology of irritable bowel syndrome in North America: a systematic review. *The American Journal of Gastroenterology*.

[3] Ringel Y, Sperber AD, Drossman DA (2001). Irritable bowel syndrome. *Annual Review of Medicine*.

[4] Gwee K-A, Lu C-L, Ghoshal UC (2009). Epidemiology of irritable bowel syndrome in Asia: something old, something new, something borrowed. *Journal of Gastroenterology and Hepatology*.

[5] Pan G, Lu S, Ke M, Han S, Guo H, Fang X (2000). An epidemiologic study of irritable bowel syndrome in Beijing—a stratified randomized study by clustering sampling. *Zhonghua Yi Xue Za Zhi*.

[6] Xiong L-S, Chen M-H, Chen H-X, Xu A-G, Wang W-A, Hu P-J (2004). A population-based epidemiologic study of irritable bowel syndrome in Guangdong province. *Zhonghua Yi Xue Za Zhi*.

[7] Chen S (1998). A study on the epidemiology and risk factors of irritable bowel syndrome. *World Chinese Journal of Digestology*.

[8] Whitehead WE, Burnett CK, Cook EW, Taub E (1996). Impact of irritable bowel syndrome on quality of life. *Digestive Diseases and Sciences*.

[9] Gralnek IM, Hays RD, Kilbourne AA, Naliboff B, Mayer EA (2000). The impact of irritable bowel syndrome on health-related quality of life. *Gastroenterology*.

[10] Sandler RS, Everhart JE, Donowitz M (2002). The burden of selected digestive diseases in the United States. *Gastroenterology*.

[11] Hulisz D (2004). The burden of illness of irritable bowel syndrome: current challenges and hope for the future. *Journal of Managed Care Pharmacy*.

[12] Hyams JS, Burke G, Davis PM, Rzepski B, Andrulonis PA (1996). Abdominal pain and irritable bowel syndrome in adolescents: a community-based study. *The Journal of Pediatrics*.

[13] Reshetnikov OV, Kurilovich SA, Denisova DV, Zavyalova LG, Tereshonok IN (2001). Prevalence of dyspepsia and irritable bowel syndrome among adolescents of Novosibirsk, western Siberia. *International Journal of Circumpolar Health*.

[14] Howell S, Talley NJ, Quine S, Poulton R (2004). The irritable bowel syndrome has origins in the childhood socioeconomic environment. *The American Journal of Gastroenterology*.

[15] Rajindrajith S, Devanarayana NM (2012). Subtypes and symptomatology of irritable bowel syndrome in children and adolescents: a school-based survey using Rome III criteria. *Journal of Neurogastroenterology & Motility*.

[16] Karabulut GS, Beşer  ÖF, Erginöz E, Kutlu T, Cokuğraş FÇ, Erkan T (2013). The incidence of irritable bowel syndrome in children using the Rome III criteria and the effect of trimebutine treatment. *Journal of Neurogastroenterology & Motility*.

[17] Miele E, Simeone D, Marino A (2004). Functional gastrointestinal disorders in children: an Italian prospective survey. *Pediatrics*.

[18] Walker LS, Lipani TA, Greene JW (2004). Recurrent abdominal pain: symptom subtypes based on the Rome II Criteria for pediatric functional gastrointestinal disorders. *Journal of Pediatric Gastroenterology and Nutrition*.

[19] Dong L, Dingguo L, Xiaoxing X, Hanming L (2005). An epidemiologic study of irritable bowel syndrome in adolescents and children in China: a school-based study. *Pediatrics*.

[20] Zhou H, Yao M, Cheng G-Y, Chen Y-P, Li D-G (2011). Prevalence and associated factors of functional gastrointestinal disorders and bowel habits in chinese adolescents: a school-based study. *Journal of Pediatric Gastroenterology and Nutrition*.

[21] Zhou H, Li D, Cheng G, Fan J, Lu H (2010). An epidemiologic study of irritable bowel syndrome in adolescents and children in South China: a school-based study. *Child: Care, Health and Development*.

[22] Zhao ZT (2000). *Methods and Application in Epidemiologic Research*.

[23] H-l JIN, Zhou D-P (1999). The psychological assessment in patients with the irritable bowel syndrome. *World Chinese Journal of Digestology*.

[24] Drossman DA, Corazziari E, Talley NJ, Thompson WG, Whitehead WE (1999). Rome II: a multinational consensus document on function gastrointestinal disorders. *Gut*.

[25] Wang K, Su LY, Zhai J, Zhu Y, Yang ZW, Zhang JS (2002). Norms of the screen for child anxiety related emotional disorders in Chinese urban children. *Chinese Journal of Clinical Psychology*.

[26] Park DW, Lee OY, Shim SG (2010). The differences in prevalence and sociodemographic characteristics of irritable bowel syndrome according to Rome II and Rome III. *Journal of Neurogastroenterology & Motility*.

[27] Thompson WG (2006). The road to Rome. *Gastroenterology*.

[28] Sperber AD, Shvartzman P, Friger M, Fich A (2007). A comparative reappraisal of the Rome II and Rome III diagnostic criteria: are we getting closer to the “true” prevalence of irritable bowel syndrome?. *European Journal of Gastroenterology & Hepatology*.

[29] Longstreth GF, Thompson WG, Chey WD, Houghton LA, Mearin F, Spiller RC (2006). Functional bowel disorders. *Gastroenterology*.

[30] Hillilä MT, Färkkilä MA (2004). Prevalence of irritable bowel syndrome according to different diagnostic criteria in a non-selected adult population. *Alimentary Pharmacology and Therapeutics*.

[31] Rasquin A, di Lorenzo C, Forbes D (2006). Childhood functional gastrointestinal disorders: child/adolescent. *Gastroenterology*.

[32] Li D-G, Liu D, Xu X-X (2005). An epidemiologic study of irritable bowel syndrome in adolescents. *Chinese Journal of Digestion*.

[33] Shen L, Kong H, Hou X (2009). Prevalence of irritable bowel syndrome and its relationship with psychological stress status in Chinese university students. *Journal of Gastroenterology and Hepatology*.

[34] Drossman DA (2006). The functional gastrointestinal disorders and the Rome III process. *Gastroenterology*.

[35] Mathias JR, Clench MH, Abell TL (1998). Effect of leuprolide acetate in treatment of abdominal pain and nausea in premenopausal women with functional bowel disease: a double-blind, placebo-controlled, randomized study. *Digestive Diseases and Sciences*.

[36] He J-Q, Wang W-A, Uh P-J, Chen W (2004). Sleep quality in patients with irritable bowel syndrome. *World Chinese Journal of Digestology*.

[37] Bengtson M-B, Rønning T, Vatn MH, Harris JR (2006). Irritable bowel syndrome in twins: genes and environment. *Gut*.

[38] Bytzer P, Howell S, Leemon M, Young LJ, Jones MP, Talley NJ (2001). Low socioeconomic class is a risk factor for upper and lower gastrointestinal symptoms: a population based study in 15 000 Australian adults. *Gut*.

[39] Kanazawa M, Endo Y, Whitehead WE, Kano M, Hongo M, Fukudo S (2004). Patients and nonconsulters with irritable bowel syndrome reporting a parental history of bowel problems have more impaired psychological distress. *Digestive Diseases and Sciences*.

[40] Locke GR, Zinsmeister AR, Talley NJ, Fett SL, Melton LJ (2000). Risk factors for irritable bowel syndrome: role of analgesics and food sensitivities. *The American Journal of Gastroenterology*.

[41] El-Salhy M (2012). Irritable bowel syndrome: diagnosis and pathogenesis. *World Journal of Gastroenterology*.

